# Successful Treatment of Chronic Osteomyelitis of the Radius

**DOI:** 10.5402/2011/154878

**Published:** 2010-12-27

**Authors:** Barry J. O'Neill, Kieran M. Hirpara, T. Kenneth Kaar

**Affiliations:** Department of Orthopaedics and Trauma Surgery, Merlin Park Hospital, Galway, Ireland

## Abstract

Chronic osteomyelitis is uncommon in the United Kingdom and Ireland. Current migration trends with an influx of people from less affluent nations may result in more cases of chronic osteomyelitis being seen in community and hospital practice. We report on a case of chronic osteomyelitis of the radius and document current treatment recommendations.

Chronic osteomyelitis is a highly debilitating condition that causes significant morbidity and can by extremely difficult to manage. Staphylococcus aureus is the most common cause of osteomyelitis, accounting for more than 50% of cases [1]. Community-acquired-methicillin resistant staphylococcus aureus (MRSA) infection in children is an increasing problem in the United Kingdom (UK) and Ireland [2] and worldwide [3]. Chronic osteomyelitis is uncommon in the UK and Ireland but has a much higher incidence in less affluent nations [4]. Current immigration trends may result in an increase in the incidence of chronic osteomyelitis in the UK and Ireland, and clinicians should be aware of current treatment practices. We present a case of chronic osteomyelitis successfully treated with debridement and antibiotics.

A 17-year-old Albanian man with chronic osteomyelitis of the right radius was transferred to Ireland by the Irish Friends of Albania charity [5]. He reports a four-year history right forearm pain with chronic discharging sinuses on the dorsal and volar aspects of the proximal forearm (Figure 1). Treatment in Albania had consisted of recurrent courses of antibiotics and drainage of soft tissue abscesses. He was systemically well but swabs of the discharge from his proximal forearm grew MRSA on culture. Clinical examination demonstrated a partial median nerve neuropathy with muscle atrophy, recurvatum deformity of the radius, and fixed flexion deformity of the elbow. Plain radiographs showed sequestrum and involucrum throughout the length of the radius, and MRI scan showed changes in the distal radial epiphysis consistent with chronic infection. The distal humerus appeared healthy. 

Surgical debridement of his right radius was performed under general anaesthetic. The radius was exposed throughout its length through a volar “Henry” approach. Both sinuses were injected with methylene blue dye and excised down onto the radius. The bony sinuses were thoroughly curetted and pulse-lavaged with five litres of normal saline. A trough of bone 5 mm wide was excised from the distal metaphysis to the bony sinus proximally. An 11 cm sequestrum of dead bone was identified and excised. Pus and gelatinous/fibrous tissue was curetted from the medullary cavity then thoroughly irrigated with 10 litres of pulsed normal saline. Twenty-two palacos-gentamicin cement beads infused with 3 g of vancomycin and 320 mg of tobramycin were fashioned, attached to a 1 nylon suture, and distributed evenly throughout the medullary canal (Figure 2). 

The proximal end of the suture and the proximal-most bead were left in the superficial soft tissues. A betadine wick was inserted over the length of the radius in the volar wound and in the dorsal sinus wound. The volar wound was closed with 3-0 nylon, and skin staples and both wounds were covered with dry dressings. Postoperatively he commenced on 1.5 g of IV vancomycin and 600 mg of rifampicin twice daily. Renal function was checked every 48 hours. Analgesia was IV morphine and oral diclofenac and paracetamol. Linezolid (600 mg twice daily for 28 days) was substituted for vancomycin after 14 days. The wounds were checked at 48 hours and showed no sign of infection. They were washed out with 5 litres of normal saline, and a small superficial betadine wick was placed. The dorsal wound was closed at 48 hours, volar wound on day 6.

Culture of the debris from the medullary canal grew MRSA. Fusidic Acid 500 mg orally three times daily was added to the antibiotic regime. Gross histopathology showed a skin ellipse with underlying firm brown tissue of 25 mm maximum depth bearing a central sinus opening connected to a tract 25 mm long. Histological analysis confirmed the presence of an inflammatory tract with no evidence of malignancy. Gross inspection of the sequestrum showed two separate portions of bony tissue 65 × 8 mm and 45 × 8 mm with firm brown tissue 25 × 12 × 4 mm, and multiple fragments of organising abscess material with spicules of calcified tissue. Culture swab of the volar wound on day 10 was sterile.

He was discharged on day 22 postoperatively and was reviewed by the senior author weekly for 6 weeks then reviewed every two weeks in the OPD and weekly in the physiotherapy department for elbow/forearm/wrist ROM exercises. He returned to Albania 5 months after his procedure, and 3 years later he remains well with no evidence of recurrence.

Although chronic osteomyelitis is rare in the United Kingdom and Ireland, increased immigration from less well-developed areas may lead to an increased incidence of this debilitating condition. Conventional treatment consists of high-dose intravenous antibiotics for at least six weeks [6]. Disadvantages of this regimen include prolonged hospital stay, cannula-site infections and thrombosis, and increased costs. Studies in some centres have shown good results with combination intravenous and oral antibiotic therapy [7]. We demonstrate cure of resistant chronic osteomyelitis of the radius with surgical debridement and combined intravenous and oral antibiotic therapy. This has obvious financial implications for the treatment of this condition. We present this case to highlight the potential increased incidence of this condition in the UK and Ireland and to emphasise that prolonged in-patient stay for intravenous antibiotic therapy is not always necessary.

## Figures and Tables

**Figure 1:**
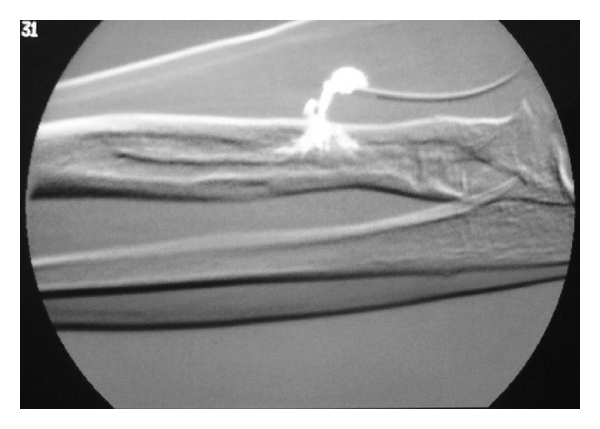
Sinogram showing fistula on volar aspect of forearm.

**Figure 2:**
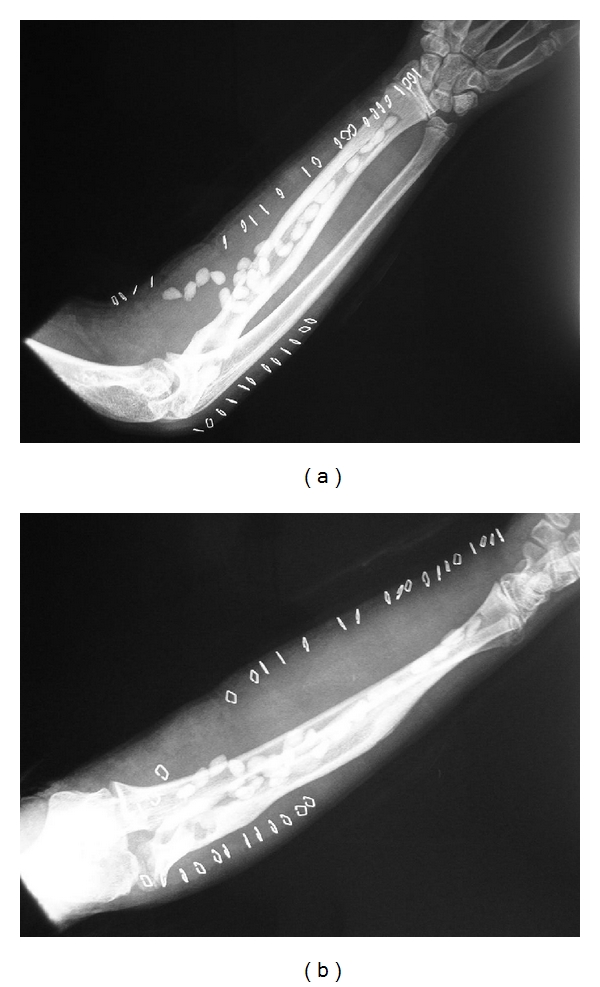
Postoperative AP and lateral radiographs of forearm.
